# Treading the Path towards Genetic Control of Snail Resistance to Schistosome Infection

**DOI:** 10.3390/tropicalmed3030086

**Published:** 2018-08-15

**Authors:** Damilare O. Famakinde

**Affiliations:** Department of Medical Microbiology and Parasitology, College of Medicine, University of Lagos, Idi-Araba, Surulere, Lagos 100254, Nigeria; damfam.joe@gmail.com; Tel.: +234-703-330-2069

**Keywords:** schistosomiasis, vector control, snail resistance, gene drive, transgenic snail

## Abstract

Schistosomiasis remains the most important tropical snail-borne trematodiasis that threatens many millions of human lives. In achieving schistosomiasis elimination targets, sustainable control of the snail vectors represents a logical approach. Nonetheless, the ineffectiveness of the present snail control interventions emphasizes the need to develop new complementary strategies to ensure more effective control outcomes. Accordingly, the use of genetic techniques aimed at driving resistance traits into natural vector populations has been put forward as a promising tool for integrated snail control. Leveraging the *Biomphalaria*-*Schistosoma* model system, studies unraveling the complexities of the vector biology and those exploring the molecular basis of snail resistance to schistosome infection have been expanding in various breadths, generating many significant discoveries, and raising the hope for future breakthroughs. This review provides a compendium of relevant findings, and without neglecting the current existing gaps and potential future challenges, discusses how a transgenic snail approach may be adapted and harnessed to control human schistosomiasis.

## 1. Introduction

It is presently more than a century since malacological discoveries established that various genera of freshwater snails (Mollusca: Gastropoda) serve as biological vectors of human diseases caused by parasitic trematodes. These findings have made the study of snail biology an important aspect of infectious disease research, particularly in tropical helminthology. Among these human snail-borne trematodiases, schistosomiasis (bilharziasis) ranks as the most important disease, afflicting more than 206 million humans [1], and being responsible for over 3.51 million disability-adjusted life years (DALYs) [2], most prominently in the tropics. Most cases of human schistosomiasis are caused by three parasitic schistosomes (blood flukes): *Schistosoma haematobium*, *S. mansoni,* and *S. japonicum* [3]. Both *S. haematobium* and *S. mansoni* are found in Africa and the Middle East; only *S. mansoni* occurs in the Americas, and *S. japonicum* is a major disease-causing species in China, Indonesia, and the Philippines [3]. However, the geographical distribution of these flukes is synchronous to, and importantly determined by, the local distribution of their snail vectors. *S. haematobium* is transmitted by *Bulinus* snails, *S. mansoni* by *Biomphalaria* snails, and *S. japonicum* by *Oncomelania* snails [4].

The human-to-snail-to-human transmission of *Schistsosoma* occurs when adult male and female living in copula within the human host mate and produce fertilized eggs. Some of the eggs are voided with urine (in *S. haematobium*) or feces (in *S. mansoni* and *S. japonicum*) into the environment. The eggs that reach the vectors’ freshwater habitats hatch and release the enclosed miracidia larvae, which swim actively to locate and infect their snail vectors. A miracidium that successfully infects a susceptible vector undergoes intramolluscan polyembryonic development to produce thousands of actively-swimming tailed cercariae larvae that emerge continuously from the snail host for the rest of the its lifetime (spanning months) [3,4,5,6]. Human infection with schistosomes is acquired through skin contact with, and subsequent penetration by, the cercariae during recreational, domestic, or occupational activities with contaminated water [5]. Following penetration, the worms transform into immature schistosomes (schistosomulae) and are carried in body circulation, from where they enter the portal veins and mature in about 5–7 weeks [3,5]. Mature worm pairs migrate to their preferred host sites—*S. mansoni* and *S. japonicum* to the mesenteric venules of the bowel or rectum, and *S. haematobium* to the venous plexus of the bladder, where they mate and the females lay eggs to repeat the cycle [4,5]. Adult schistosomes have an average lifespan of 3–10 years, but they may also live as long as 30–40 years in their human hosts [3,4,5]. The eggs are highly immunogenic and are majorly responsible for disease outcomes by triggering localized pathologic reactions within the human host [4,7,8]. Although human infection with *Schistosoma* species may cause non-specific but incapacitating systemic morbidities such as malnutrition, anemia, and impaired physical and cognitive development in children, poor birth outcomes in infected pregnant women, and neurological aberrations, *S. haematobium* is specifically responsible for urogenital pathologies, while other *Schistosoma* species majorly cause gastrointestinal complications, but also hepatosplenic enlargement, ascites, and portal hypertension in advanced cases [3,7,9,10]. Again, there is growing evidence that female urogenital schistosomiasis poses an increased risk of HIV transmission and/or progression [11,12,13].

Taking a leap towards the beginning of the end human schistosomiasis requires an integrated control approach that cuts across both the vector and the human cycles. Current strategy in the fight against the disease co-implements ongoing preventive chemotherapy through mass drug administration (MDA), with complementary public-health interventions. This approach, as defined by WHO/AFRO, is known as PHASE—preventive chemotherapy, health education, access to clean water, sanitation improvement, and environmental snail control and focal mollusciciding [14]. Recent efforts made to evaluate the degree of importance of snail control in schistosomiasis elimination [15,16,17,18] clearly showed that sustainable snail control is pivotal in achieving targeted disease elimination. This is especially true in the present era of highly challenging anti-schistosome vaccine development, as well as the monochemotherapeutic availability of praziquantel and its feared resistance by schistosomes [19,20,21]. Strategies currently in use for controlling schistosomiasis snail vectors are: biocontrol using competitors or predators, modification of snail habitats, and application of molluscicides. These approaches, used either singly or in combination, have evidently contributed to many successful schistosomiasis control efforts in different localities and countries [15,22,23,24,25,26,27]; however, each approach is not without limitations [24]. The application of chemical molluscicides has been mostly exploited. Among other chemical molluscicidal agents, niclosamide has a long track record of being successful against snail hosts, and is often regarded as the molluscicide of choice. Nevertheless, apart from its expensiveness, toxicity of niclosamide to a variety of non-target aquatic life forms (plants, invertebrates and vertebrates including amphibians) has led to its decreased acceptability. Again, the inability of niclosamide to prevent snail recolonization, especially in large permanent water bodies, necessitates repeated applications that result in high cost [24,28,29,30].

In view of the present challenges facing schistosomiasis control efforts, coupled with the endorsement by the World Health Assembly Resolution 65.21 to take full advantage of non-drug-based interventions to prevent schistosomiasis transmission [31], it will be timely to adapt new strategies in order to interrupt snail-mediated schistosome transmission, and thus, forestall human infection. The use of genetic techniques to manipulate snail vectors of schistosomiasis has long been stressed as a novel biocontrol strategy with the potential to constitute an important complementary tool for transmission reduction or breaking. Embracing all the means to actualize this potential, studies unraveling the complexities of the vector biology and those exploring the molecular underpinnings of snail resistance/susceptibility to schistosome infection have been expanding in various breadths, generating many significant discoveries and raising the hope for future breakthroughs. The aim of this review is to provide a compendium of relevant findings, and discuss how transgenic snail approach may be adapted and harnessed to control human schistosomiasis.

## 2. Biology of Snail Resistance/Susceptibility to *Schistosoma* Infections–Major Exploits so Far

The first groundbreaking discovery on the identification of intermediate snail hosts of schistosomes was made by Miyairi and Suzuki, who observed stages of *S. japonicum* in *Oncomelania* snails in Japan in 1913 [32,33]. This was followed by the achievements of Robert Leiper, who also demonstrated the complete life cycles of *S. haematobium* and *S. mansoni* in their respective snail hosts in Egypt [34,35]. Subsequent to these watershed moments in the long history of schistosomiasis, investigations on the interactions between schistosomes and their snail vectors became kinetic. The genetic study of snail-schistosome compatibility was pioneered by Newton [36,37], who demonstrated that susceptibility of snail vectors to *Schistosoma* infections is fundamentally genetic and a heritable character. This was later underscored by other investigators who revealed that resistance character, which is acquired at the maturity phase in the adults of resistant snail stocks, is monogenic, dominant, and heritable by a simple Mendelian pattern of inheritance [38,39,40,41]. This genetic dominance of the resistance trait has been confirmed by various crossbreeding experiments in *Biomphalaria* species [42,43,44,45,46]. Be that as it may, Rosa et al. [45] showed that resistance in *B. tenagophila* is determined by two dominant genes. In contrast, in juvenile *B. glabrata*, resistance is a complex trait governed by a minimum of four genes, each having multiple alleles (alternative forms of the same gene) [40,47]. From these various lines of evidence, it could be understood that genetic determinism of resistance is governed by a single major locus (position of a particular gene or allele on a chromosome) to a potentially high number of loci, and snails with significantly increased resistance could be artificially selected in the laboratory; meanwhile, molecular markers mapped to resistance could be identified in genetic crosses.

Thus far, work has been done most extensively using the *Biomphalaria*-*Schistosoma* model, and has led to the nomenclature of some stocks known for resistance (e.g., pigmented BS-90 [48], black-eye 10-R2 [49], and 13-16-IR [50]) or susceptibility (e.g., the albino M-line and NMRI [51], and BB02 [52]) to *S. mansoni* infection, which are now maintained in the laboratory for research purposes. In contrast to the 10-R2 and 13-16-IR strains, however, BS-90 demonstrates unflinching resistance stability, irrespective of age (juvenile or adult), under laboratory conditions [40,53].

A major physiological determinant of snail resistance/susceptibility to infections, which is also under genetic influence, is the snail internal defense system (IDS). The IDS comprises the cellular elements (hemocytes) and the humoral (plasma) factors of the hemolymph that work independently or in concert to recognize, encapsulate, kill, and clear intruding trematodes [6,54,55,56]. Establishment of the *B. glabrata* embryonic (*Bg*e) cell line in 1976 [57] provided an enabling avenue for investigators to delve into the molecular and cellular aspects of the complex snail immune functions against schistosomes by using an *in vitro* culture model, rather than using the whole intact animal, which could have resulted in a rudimentary understanding of the complex biological events. Moreover, major advances in *Biomphalaria* omic studies, such as the recent availability of the whole genome sequence of *B. glabrata* [58], provide a useful resource in deciphering complex functions of the snail biology that were previously obscure. Using various strain and species combinations of the *Biomphalaria*-*Schistosoma* model system, robust molecular studies have been carried out, leveraging various techniques to identify and characterize endogenous effector protein/gene candidates that are functional in the snail internal defense machinery against schistosomes. Table 1 below presents a synopsis of various endogenous factors that have been implicated in *Biomphalaria* resistance to schistosomes.

## 3. Transgenic Snail Methods for Schistosomiasis Control

The use of genetically engineered vectors to either suppress (reduce) or modify (replace) the natural populations of the biological vectors of some globally important infectious diseases has been a convincing concept that is now on the verge of deployment to control disease transmission. This rapidly emerging genetic control approach is distinguished from other biocontrol strategies (such as the use of natural parasitoids, predators, competitors, and infectious microbial agents), as it is mating-based, highly species-specific, and capable of being transmitted or inherited vertically [83]. In this context, suppression or elimination of natural local vector populations could be achieved by releasing transgenic vectors carrying sterile or detrimental characters into the local populations, while the population modification approach requires the release of transgenic pathogen-resistant vector strains or species. Depending on their characteristics or specific configurations, genetic methods for transgenic vector control may be self-limiting with transient persistence among subsequent vector generations unless replenished by repeated release of engineered vectors, or self-sustaining with indefinite persistence from the initial target population to the subsequent ones [83]. With revolutions in biotechnology, the use of gene drive systems (self-sustaining, selfish genetic elements that are inherited by progenies at frequencies largely exceeding those expected in Mendelian inheritance) has become an attractive method for vector control applications, as gene drivers are invasive wherever present, and so may overcome some evolutionary constraints [83,84].

**Figure 1:**
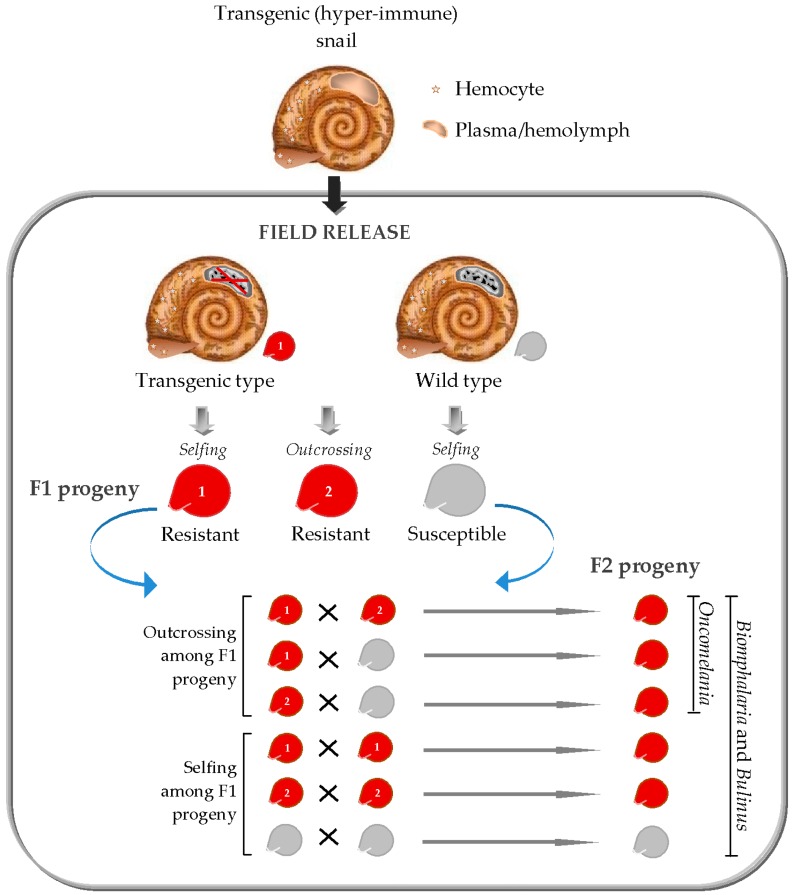
Transgenic snail system for field control of schistosomiasis transmission.

As first proposed in 1958 [85], much emphasis has been placed on the use of genetic biocontrol for schistosomiasis vector control. To select a suitable transgenic vector method for schistosomiasis control however, the basics of the biology of the snail vectors must be taken into account. Although a wealth of genetic studies has been centered on the production of transgenic mosquito vectors of human diseases, biological differences between these dipterans and schistosomiasis molluscan vectors create the need for selective transgenic vector strategies for schistosomiasis control application. For example, unlike in mosquitoes where only females are capable of transmitting pathogens, *Biomphalaria* and *Bulinus* are hermaphrodites capable of self- or cross-fertilization [86], and all mating types serve as hosts for schistosomes. *Oncomelania* has separate sexes [86] but nevertheless, both sexes can also carry *S. japonicum*, only perhaps at varying degree of susceptibility [87,88]. These biological peculiarities render population reduction strategies unsuitable for genetic control of the snail hosts. This is because the newly released transgenic snails carrying harmful characters would remain susceptible to *Schistosoma* infections, therefore intensifying parasite transmission. Consequently, present focus in the genetic control of schistosomiasis vectors is set on strategies to modify the natural snail populations. According to Hubendick [85], population of the resistant strains can displace that of the susceptible ones in the field by natural selection. Although this scenario is plausible, it can be realized only through the application of self-sustaining transgenic vector systems (Figure 1).

Promoted by its advantages over other gene drive systems—such as transcription activator-like effector nucleases (TALENs) and zinc finger nucleases (ZFNs)—used in genome editing technology, and over other genetic techniques such as RNA interference (RNAi) [84,89,90], the recently-discovered CRISPR/Cas9 (clustered regularly-interspaced short palindromic repeats/CRISPR-associated protein 9) system has flowered, and is being widely used in current research trials and applications to modify genome sequences in diverse species spanning microbes, plants, animals, to even humans [89,90,91,92,93]. In parallel, the use of CRISPR/Cas9 to drive anti-*Schistosoma* effector genes into the genome of naturally susceptible snail strains is being envisioned, and has become an important subject in current discussions [17,75,94,95]. Fascinatingly, a proof-of-concept study [96] has demonstrated the possibility of CRISPR/Cas9-mediated gene editing in molluscs; indeed, more insights into the possible ways by which this may be achieved for schistosomiasis vector control, as well as the current and potential future challenges, will constitute a key guide for the scientific community in the appropriate fostering of this tantalizing approach in snail molecular research.

**Figure 2:**
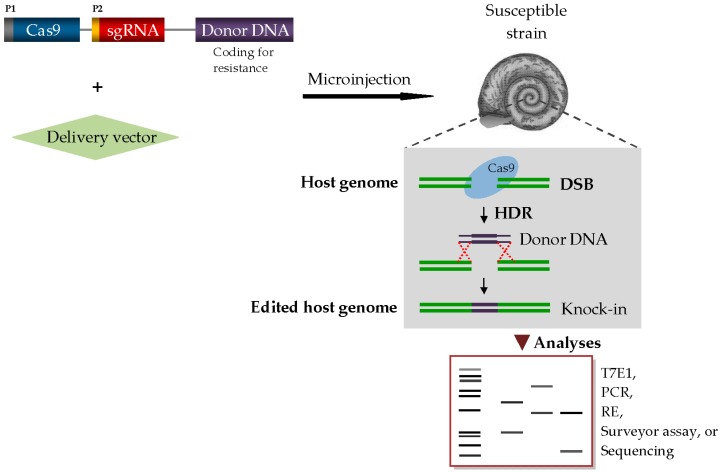
A schematic representation of CRISPR/Cas9 genome editing system in a snail vector of schistosome. Abbreviations: DSB, double-strand break; HDR, homology-directed repair; P1 & P2, promoters; PCR, polymerase chain reaction; RE, restriction enzyme; T7E1, T7 endonuclease I.

The three basic requirements for a CRISPR-based precise gene knock-in editing are Cas9 endonuclease, single-guide RNA (sgRNA), and repair template DNA (donor). The Cas9 enzyme combs through the genome of the host organism, acting as the ‘molecular scissors’ that cuts a specific DNA sequence at a genomic locus. The sgRNA (~20 nucleotides) is designed to match and target the desired DNA sequence to be deleted, while the donor DNA provides a template for genomic repair of the cleaved locus [92,97]. In the case of schistosomiasis snail vectors, Cas9-mediated introgression of refractoriness into susceptible strains will require an engineered donor DNA encoding a locus known to confer resistance. The anti-*Schistosoma* donor DNA can be tightly anchored to the Cas9/sgRNA complex, and the entire cassette is co-injected with a viral vector, such as lentivirus [98], into the early single-cell stage embryos of the snail vectors (Figure 2). In essence, the viral vector ensures safe and effective delivery of these components into the nuclei of the target cells. Suitable sites for the driver–cargo system injection may include the ovotestis of *Biomphalaria*/*Bulinus* snails and the ovary of female *Oncomelania* snails. In *Biomphalaria*, the ovotestis is located at the tip of the shell spire [99] and the driver–cargo system insertion into the ovotestis will be more appealing in the albino strain, as the transparency of the snail shell allows easy visibility of internal organs. Further analyses to assess targeting efficiency or screen for transgenic mutants among progenies may be done by T7 endonuclease I (T7E1) assay, restriction enzyme assay, next generation sequencing or direct PCR assay as applicable (Figure 2).

To date, the main genetic loci that have been identified in association with *B. glabrata* resistance to schistosome are *Sod1* and *RADres* (a restricted-site associated DNA-determined resistance locus) [50,68,69], and a *GRC* (Guadeloupe Resistance Complex) genomic region (<1 Mb) [69]. In combination with other known and yet unknown resistance genes, *Sod1* and *RADres* occupy haplotype blocks of >2 Mb genomic region [69,100]. Although putative functional gene candidates have been identified in the *GRC* region [75,95], the *Sod1* and *RADres* regions appear to demonstrate a wider spectrum of snail resistance [69]. Nevertheless, there is still a need to further narrow down these regions to the embedding causative genes, and to understand their immune stability and functions under different genetic backgrounds and environmental conditions.

## 4. Further Considerations

An early investigator [101] stated that the genetic factors controlling snail insusceptibility to schistosomes must first be clarified, and snail strains ferrying only refractory traits must be developed before we can gainfully engage genetic control methods. The first criterion has largely been met through relentless research unveiling resistance-determining proteins and genes. Despite these advances, current stumbling blocks involve developing snail strains that are reliably recalcitrant to schistosome infection. One major bottleneck is the highly variable strain-by-strain interaction—compatibility polymorphism—that is well-documented to occur in snail-schistosome systems [102,103]. As a consequence, developing a transgenic target for individual strain-to-strain combinations becomes cumbersome, but can be circumvented only if genetic loci with wide-spectrum resistance activities conserved across various strain-to-strain combinations could be identified and characterized. The BS-90 strain of *B. glabrata* (isolated in Salvadore, Brazil) has been bred in the laboratory for many years and has been shown to be steadily resistant; however, its relative performance in the field remains unpredictable. A tenable reason for this is that generations of the laboratory-bred strains are poor representatives of the genetic variations that actually occur in the original wild populations [103]. Another caveat in the future use of either the resistant BS-90 or transgenic snail strains is global warming, characterized by an increasing earth’s average surface temperature. In sharp contrast to what was earlier known, Knight et al. [78] showed that snail resistance to schistosomes is also temperature-dependent, and even the naturally resistant BS-90 strain could be rendered susceptible at 32 °C. Other local environmental factors such as altitude, water level, soil, and vegetation may also cause differential gene expression and regulation among snails of the same species as a result of local adaptation mechanisms [104].

Organism biodiversity and signatures of interactions between other organisms and the snail vectors living in the same habitat may also impact the outcome of transgenic snail application. In an ecological milieu where natural predators [e.g., *Macrobrachium vollenhovenii* (a freshwater prawn), *Procambarus clarkia* (a freshwater crayfish), *Marisa cornuarietis* (an ampullarid snail), and cichlid fishes such as *Trematocranus placodon* and *Geophagus brasiliensis*] or competitors [e.g., thiarid snails such as *Melanoides tuberculata* and *Tarebia granifera*] of the snail vectors of schistosomiasis [4,24] exist in meaningful abundance, there is a possibility that the population of the released transgenic snails becomes reduced below levels required to displace that of the naturally susceptible vectors as a result of a more biased killing/eating of the transgenic snails (and eating of their egg masses) or deprivation of resources. When such a scenario operates, the resistance effect tapers off. Given this contingency, the release of transgenic snails may be chosen only in lieu of introducing predators or competitors of snail vectors; co-implementation of both methods in the same freshwater focus may not always complement the transgenic snail approach. In foci where populations of predators or competitors already occur in significant abundance, one-off niclosamide application prior to the release of transgenic snails may offer a more palatable approach in reducing the probability of diluted effect of the transgenic snail release. These phenomena highlight the importance of sampling water habitats for species diversity prior to, and periodically after, releasing transgenic snails.

The merits of using schistosome-resistant transgenic snails beat the limitations of other biological and environmental interventions. For instance, populations of molluscivorous fishes and prawns large enough to eat the snail vectors may rapidly diminish due to indiscriminate fishing by residents of communities where schistosomiasis is endemic, since these molluscivores are also a major source of food for humans. Moreover, introduction of competitor species of snails could greatly endanger agriculture and the ecosystem. On the other hand, environmental modifications (such as removal of vegetation on which the snail vectors feed, lining canals with cement, or draining water habitats) are very expensive and impractical for resource-constrained areas. Meanwhile, vegetation removal poses an increased risk of infection to workers who may not have protective tools [24]. Generally, however, certain issues concerning the use of gene drive systems have come into view. The most important of all include potential off-target mutations that may result in unpredictable effects, development of drive resistance in populations, fitness and competitiveness of released strains compared to wild populations, and possible difficulty in the containment, reversal, or adjustment of gene drive spread [83,84,105]. Nevertheless, it is somewhat relieving that a good number of these limitations can feasibly be overcome through the meticulous design of more specific sgRNAs, and development of reversal drive systems [84,89,91,92,97,105]. Moreover, the majority of the current issues regarding the application of gene drives for the control of disease vectors arose from studies focusing on mosquitoes, implying that some of the risk issues, such as vector dispersal beyond intended political boundaries [84], may be of lesser concern in other non-insect vector control systems. Conversely however, the significant body of research on mosquitoes may have also overcome some series of technical challenges that may remain unresolved for other disease vectors.

Should a breakthrough on the use of CRISPR-based vector control occur, the fine line between mating/reproductive biology of *Oncomelania* and that of *Bulinus* or *Biomphalaria*, as well as the varying degree of selfing among species of the hermaphroditic (*Bulinus* and *Biomphalaria*) snail vectors, will also have important implications in schistosomiasis snail control application. As shown in Figure 1, CRISPR/Cas9-driven resistance traits may spread more rapidly among successive progeny of *Oncomelania* (being a dioecious outcrossing vector) than in *Bulinus* and *Biomphalaria* snail vectors. More precisely, in the two latter snail vectors, gene drive approach may not be effective in predominantly selfing species, such as *Bulinus truncatus*, *Bulinus forskalii*, and *Biomphalaria pfeifferi*.

## 5. Conclusions

The prospective use of genetically manipulated vectors to stop the spread of vector-borne diseases maintains its impressiveness and is awaited by the scientific community. In fast-tracking sustainable schistosomiasis elimination, the use of CRISPR-based vector modification strategy appears fascinating and potentially effective. However, this approach is currently still underdeveloped in snail molecular research. Finding the pertinent missing pieces in our jigsaw of knowledge of schistosome/snail biology, and identifying ways to bypass potential future challenges, are requisites for achieving this promising snail control strategy. Finally, the use of schistosome-resistant transgenic snails may have the propensity to singly interrupt schistosomiasis transmission when only outcrossing vector species are present, but in foci where both predominantly selfing species and outcrossing species of *Bulinus* or *Biomphalaria* snails coexist, the integration of additional suitable snail control methods will provide a way of complementing this genetic control method for more effective outcomes.

## Figures and Tables

**Table 1:** Putative genes and proteins conferring *Biomphalaria* resistance to *Schistosoma* infection.

Resistance Factor	Snail spp.	*Schistosoma* spp.	Function	Reference(s)
40S ribosomal protein S9	*B. glabrata*	*S. mansoni*	Protein translation in hemocytes.	[59]
*Bg*AIF	*B. glabrata*	*S. mansoni*	Modulates hemocyte activation.	[60]
*Bg*GRN	*B. glabrata*	*S. mansoni*	Production of adherent hemocytes.	[61]
*Bg*MIF	*B. glabrata*	*S. mansoni*	Induces hemocyte proliferation.	[62]
*Bg*TLR	*B. glabrata*	*S. mansoni*	Parasite recognition and activation of effector functions.	[63]
Biomphalysin	*B. glabrata*	*S. mansoni*	Binds to the sporocyst surface and lyses it.	[54,64]
Cathepsin B	*B. glabrata*	*S. mansoni*	Lysis of encapsulated sporocyst.	[65]
Cathepsin L	*B. glabrata*	*S. mansoni*	Lysis of encapsulated sporocyst.	[66]
Copine 1	*B. glabrata*	*S. mansoni*	Involves in signaling processes.	[66]
CREPs	*B. glabrata*	*S. mansoni*	Pattern recognition receptors/adhesion proteins.	[67]
Cu/Zn SOD (SOD1)	*B. glabrata*	*S. mansoni*	Catalyzes the production of H_2_O_2_ which is cytotoxic to sporocyst.	[50,68,69]
Cystatin 2	*B. glabrata*	*S. mansoni*	Protease inhibitor.	[70,71]
Cytidine deaminase	*B. glabrata*	*S. mansoni*	Nucleobase, nucleoside, nucleotide, and nucleic acid metabolism.	[47]
Cytochrome b	*B. glabrata*	*S. mansoni*	Mitochondrial respiration.	[70]
Cytochrome C oxidase subunits	*B. glabrata*	*S. mansoni*	Mitochondrial respiration.	[70,71]
Dermatopontin2	*B. glabrata*	*S. mansoni*	Participates in hemocyte adhesion and encapsulation responses.	[59,67,70]
Elastase2	*B. glabrata*	*S. mansoni*	Lysis of encapsulated sporocyst.	[66,70]
Elongation factors 1α & 2	*B. glabrata*	*S. mansoni*	Transcription enzymes (bind t-RNA to ribosomes).	[59,67]
Endo-1,4-β-glucanase	*B. glabrata*	*S. mansoni*	Carbohydrate metabolism.	[70]
Ferritin	*B. glabrata*	*S. mansoni*	Stores and transport iron in non-toxic form.	[70,71]
FREP1, 2, 3 & 12	*B. glabrata*	*S. mansoni*	Pattern recognition receptors/adhesion proteins.	[67,70,72,73]
Fribillin	*B. glabrata*	*S. mansoni*	Participates in hemocyte adhesion and encapsulation responses.	[70]
GlcNAc ^↓^	*B. tenagophila*	*S. mansoni*	Increases hemocyte binding to sporocyst.	[74]
GPCR kinase 2	*B. glabrata*	*S. mansoni*	Signal transduction.	[70]
Grctm6	*B. glabrata*	*S. mansoni*	Modulates cercarial shedding.	[75]
GREPs	*B. glabrata*	*S. mansoni*	Pattern recognition receptors/adhesion proteins.	[67]
GSTs	*B. glabrata*	*S. mansoni*	Prevent cellular damage to the hemocytes.	[70]
Hsp40, 60 & 70 ^#^	*B. glabrata*	*S. mansoni*	Housekeeping cell repair activities.	[66,67,70,76,77,78]
Importin 7	*B. glabrata*	*S. mansoni*	Involves in signaling processes.	[66]
Inferred phagocyte oxidase	*B. glabrata*	*S. mansoni*	Production of superoxide anions.	[60]
Interleukin 1	*B. glabrata*	*S. mansoni*	Stimulates hemocyte defense response.	[79]
LPS-binding protein	*B. glabrata*	*S. mansoni*	Adhesion protein.	[67]
Matrilin	*B. glabrata*	*S. mansoni*	Participates in hemocyte adhesion and encapsulation responses.	[59,70]
Metalloproteases	*B. glabrata*	*S. mansoni*	Tissue morphogenesis/remodeling.	[67]
MPEG 1	*B. glabrata*	*S. mansoni*	Participates in hemocyte defense responses.	[47]
Neo-calmodulin	*B. glabrata*	*S. mansoni*	Cacium signaling and homeostasis.	[67]
NF-kB	*B. glabrata*	*S. mansoni*	Downstream transcription in the TLR pathway.	[59,63,70,80]
NADH dehydrogenase subunis	*B. glabrata*	*S. mansoni*	Mitochondrial respiration.	[70]
Peroxiredoxines 1 & 4	*B. glabrata*	*S. mansoni*	Neutralize ROS and RNS that can damage cellular functions.	[60,81]
PGRP 1	*B. glabrata*	*S. mansoni*	Pattern recognition receptor.	[70]
PKC receptor	*B. glabrata*	*S. mansoni*	Signal transduction.	[47]
TEPs	*B. glabrata*	*S. mansoni*	Pattern recognition receptors/adhesion proteins.	[67]
TNF-α	*B. glabrata*	*S. mansoni*	Stimulates hemocyte defense response.	[82]

Symbols: ^↓^ in lower concentrations; ^#^ contrasting reports (see [67,78] for some details). Abbreviations: *Bg*AIF, *B. glabrata* allograft inflammatory factor; *Bg*GRN, *B. glabrata* granulin; *Bg*MIF, *B. glabrata* macrophage migration-inhibitory factor; *Bg*TLR, *B. glabrata* Toll-like receptor; CREP, C-type lectin-related protein; Cu/Zn SOD, copper/zinc superoxide dismutase; FREP, fibrinogen-related protein; GlcNac, N-acetyl-D-glucosamine; GPCR, G-protein coupled receptor; Grctm, Guadeloupe resistance complex transmembrane; GREP, galectin-related proteins; GSTs, glutathione-S-transferases; H_2_O_2_, hydrogen peroxide; Hsp, heat shock protein; LPS, lipopolysaccharide; MPEG, macrophage expressed gene; NADH, reduced nicotinamide adenine dinucleotide; NF-kB, nuclear factor kappa B; PKC, protein kinase C; PGRP, peptidoglycan recognition protein; RNS, reactive nitrogen species; ROS, reactive oxygen species; t-RNA, transfer ribonucleic acid; TEP, thioester-containing protein; TNF-α, tumor necrosis factor-alpha.
